# Sheng Jiang San, a traditional multi-herb formulation, exerts anti-influenza effects in vitro and in vivo via neuraminidase inhibition and immune regulation

**DOI:** 10.1186/s12906-018-2216-7

**Published:** 2018-05-08

**Authors:** Tianbo Zhang, Mengjie Xiao, Chun-Kwok Wong, Ka-Pun Chris Mok, Xin Zhao, Huihui Ti, Pang-Chui Shaw

**Affiliations:** 1 School of Life Sciences, The Chinese University of Hong Kong, Shatin, N.T, Hong Kong, SAR 999077 People’s Republic of China; 2 State Key Laboratory of Respiratory Disease, Guangdong Provincial Key Laboratory of Molecular Target & Clinical Pharmacology, School of Pharmaceutical Sciences and The Fifth Affiliated Hospital, Guangzhou Medical University, Guangzhou, 510632 People’s Republic of China; 3 Guangzhou Institutes of Biomedicine and Health, Chinese Academy of Science, Guangzhou, 510632 People’s Republic of China; 4 Department of Chemical Pathology, The Chinese University of Hong Kong, Prince of Wales Hospital, Shatin, N.T, Hong Kong, SAR 999077 People’s Republic of China; 5 Institute of Chinese Medicine and State Key Laboratory of Phytochemistry and Plant Resources in West China, the Chinese University of Hong Kong, Shatin, N.T, Hong Kong, SAR 999077 People’s Republic of China; 6 HKU-Pasteur Research Pole, School of Public Health, Li Ka Shing Faculty of Medicine, The University of Hong Kong, Pok Fu Lam, Hong Kong, SAR 999077 People’s Republic of China; 7 Li Dak Sum Yip Yio Chin R & D Centre for Chinese Medicine, The Chinese University of Hong Kong, Shatin, N.T, Hong Kong, SAR 999077 P. R. China

**Keywords:** Sheng Jiang san, Anti-influenza activity, Neuraminidase inhibition, Immune regulation

## Abstract

**Background:**

Sheng Jiang San (SJS), a multi-herb formulation, is used in treating high fever, thirsty and anxiety in ancient China and it is sometimes used to treat seasonal influenza nowadays. However, there is no evidence-based investigation and mechanism research to support the anti-influenza efficacy of SJS. This study aims at evaluating the anti-influenza effect of SJS and investigating its possible mechanism.

**Methods:**

The inhibitory effect of SJS against different influenza virus strains on MDCK cells was examined. Influenza virus infected BALB/c mice were employed to evaluate the efficacy as in vivo model. Mice challenged with A/PR/8/34 (H1N1) were orally administrated 1 g/kg/day of SJS for seven days and monitored for 14 days. The survival rate, body weight changes, lung index, lung viral load, histopathologic changes and immune regulation of the mice were measured. The underlying anti-influenza virus mechanism of SJS was studied by a series of biological assays to determine if hemagglutinin, ribonucleoprotein complex or neuraminidase were targets of SJS.

**Results:**

Results showed SJS exerted a broad-spectrum of inhibitory effects on multiple influenza strains in a dose-dependent manner. IC_50_ of SJS against A/WSN/33 (H1N1) was lower than 35 μg/ml. SJS also protected 50% of mice from A/PR/8/34 (H1N1) infection. The lung index and the lung viral load of SJS treated mice were significantly decreased compared with untreated mice. Meanwhile, SJS targeted on neuraminidase of influenza virus as SJS at 2 mg/ml inhibited 80% of neuraminidase enzymatic activity. SJS also significantly down-regulated TNF-α and up-regulated IL-2 of influenza virus induced mice.

**Conclusions:**

Thus, SJS is a useful formulation for treating influenza virus infection.

**Electronic supplementary material:**

The online version of this article (10.1186/s12906-018-2216-7) contains supplementary material, which is available to authorized users.

## Background

Influenza is a contagious respiratory illness causing seasonal epidemics and occasional pandemics. The death toll of influenza epidemics is between 250,000 to 500,000. The frequent reassortment of influenza virus may cause high mortality and over-burden the healthcare system [1]. For example, the outbreak of 2009 H1N1 pandemic (swine flu) caused around 185,000 of people death [2]. The most recent 2013 H7N9 is the largest annual epidemics in China also caused significant morbidity and mortality [3].

To date, two classes of anti-influenza drugs are commonly used [4]. One consists of inhibitors of the M2 ion channel, such as amantadine and rimantadine. Treatment with these drugs results in the emergence of resistant strains thus it is not recommended for general use. The other consists of neuraminidase inhibitors, such as oseltamivir, zanamivir, laninamivir and peramivir. In addition, ribavirin and favipiravir (T-705) show anti-viral RNA polymerase effect [5, 6]. However, resistance against these drugs has already emerged in recent years [7]. These highlight the urgent need for new anti-influenza agents.

Traditional herbal medicine remains an under-explored, yet potentially fruitful basis for antiviral discovery [8]. In ancient China, some Chinese prescriptions were used to treat Wen Bing (Warm Disease), which is considered as influenza in modern time, with influenza-like symptoms, such as high fever, thirsty and anxiety [9]. To date, these prescriptions are still used in clinics by traditional Chinese medical practitioners. Also, in South China, multi-herb drink or “cooling herbal tea” is a convenient folk treatment against normal cold or mild influenza [10]. However, the efficacy of most of these products has not been vigorously tested. There is no conclusive experimental evidence to support the clinical efficacies of these prescriptions in treating influenza. Nevertheless, researchers have begun to evaluate the therapeutic values and underlying mechanism of selected prescriptions, including Chinese patent drugs and traditional Chinese prescriptions [11–13]. For example, Lianhua Qingwen capsule [14] was shown to have broad-spectrum efficacy on a number of influenza virus strains, through regulating the immune responses after virus infection. Kang Bing Du oral liquid [15] was found to reduce the susceptibility to influenza virus via mitochondrial antiviral signaling.

Sheng Jiang San (SJS) is a famous Chinese prescription that was originally recorded in a Traditional Chinese Medicine Classic Shanghan Wenyi Tiaobian of Qing Dynasty. SJS is composed of *Rhei Radix et Rhizoma, Bombyx Batryticatus, Cicadae Periostracum* and *Curcumae Longae Rhizoma* in a ratio of 4:2:1:3 (*w*/w/*w*/w). It has been prescribed in treating “Warm Disease”. In modern time, traditional Chinese medical practitioners use it to treat seasonal influenza. However, there is no proper statistics on its clinical efficacy and revelation of the anti-influenza virus mechanism. Our preliminary test showed that it could indeed inhibit influenza A/WSN/33 (H1N1) in cell culture. As a contribution to increase the clinical value and the modernization of Chinese medicine, we set forth to examine the influenza inhibitory effect of SJS.

Currently, influenza virus infected mouse model is frequently used to test the in vivo influenza therapeutic efficacy of a drug [11–16]. In this study, except examining the inhibitory effect of SJS against different influenza virus strains on Madin-Darby canine kidney (MDCK) cells, we also used influenza virus infected BALB/c mice as an in vivo model to investigate the therapeutic action of SJS. The underlying anti-influenza virus mechanisms were studied by a battery of biological assays, which include viral absorption and release, and the function of viral polymerase complex.

## Methods

### Reagents

*Rhei Radix et Rhizoma, Bombyx Batryticatus, Cicadae Periostracum* and *Curcumae Longae Rhizoma* were purchased from Zisun Chinese Pharmaceutical Co., Ltd. (Guangzhou, China). Standard compounds of rhein, chrysophanol, emodin, aloe emodin and curcumin were purchased from Chengdu Pufeide Biotechnology Co., Ltd. (Chengdu, China). Oseltamivir was purchased from Yichang Changjiang Pharcaceutical Co., Ltd. (Wuhan, China). Minimum essential medium (MEM), Dulbecco’s modified eagle medium (DMEM) and fetal bovine serum (FBS) were purchased from Life Technologies (Gibco, NY, USA). Neuraminidase inhibitors screen kit (no. P0309) was purchased from Beyotime Institute of Biotechnology Co., Ltd. (Shanghai, China). Chicken erythrocytes were purchased from Lampire Biological Laboratories (PA, USA). Tolylsulfonyl phenylalanyl chloromethyl ketone (TPCK) treated-trypsin was purchased from Sigma-Aldrich (St. Louis, MO, USA). Mouse TNF-α, IFN-α and IL-2 Enzyme-linked immunosorbent assay (Elisa) kit were purchased from Invitrogen (Carlsbad, CA, USA). Water used in this study was purified by a Milli-Q system (Millipore, MA, USA). All culture plates were obtained from Greiner (Cellstar, Germany).

### Preparation of SJS extract

The identities of *Rhei Radix et Rhizoma, Bombyx Batryticatus, Cicadae Periostracum* and *Curcumae Longae Rhizoma* were confirmed by an expert at the Institute of Chinese Medicine, The Chinese University of Hong Kong, by referring to their organoleptic characteristics. The voucher specimens were kept at Li Dak Sum Yip Yio Chin R & D Centre for Chinese Medicine, The Chinese University of Hong Kong. The aqueous extract of SJS was prepared by boiling the herbs at 4:2:1:3. The four ingredients in proportion were boiled twice with deionized water for 1 h each time. The aqueous extract was filtered and concentrated by a rotary evaporator under vacuum in a 60 °C water bath. Then the concentrated extract was lyophilized into powder under vacuum of 105 × 10^− 3^ mbar and − 40 °C. The freeze-dried powder was dissolved in culture medium or water before used.

Quality control is important in Chinese prescription, as the consistency will affect the repeatability of experiments and clinical efficacy. In light of this, a large amount of freeze-dried powder of SJS was prepared only once for studies to avoid composition differences between different batches of herbs. SJS powder was analyzed by high-performance liquid chromatography (Additional file 1) and the chemical profile is shown in Additional file 2: Figure S1. By comparing with reference compounds, rhein, chrysophanol, emodin, aloe emodin and curcumin were found.

### Cells, viruses and animals

MDCK cells and human embryonic kidney 293 T (293 T) cells were obtained from American Type Culture Collection and routinely cultured in MEM and DMEM, respectively, supplemented with 10% FBS and incubated at 37 °C with 5% CO_2_. Influenza A/WSN/33 (H1N1) (WSN), A/PR/8/34 (H1N1) (PR8), A/GZ/GIRD07/09 (H1N1), A/HK/8/68 (H3N2), A/Aichi/2/1968 (H3N2), A/HK/Y280/97 (H9N2), A/China/24/96 (H7N3), B/Lee/1940 (Flu B) were provided by Dr. Zifeng Yang (Guangzhou Institute of Respiratory Disease, China). All in vitro tests were performed in class II biosafety cabinet.

Specific-pathogen-free Balb/c mice weighing 14–16 g were used in this study. Mice were obtained from Guangdong Medical Laboratory Animal Center (Guangzhou, China). The animal experiments were carried out according to the Guidelines of Guangdong Regulation for the Administration of Laboratory Animals. The mice were kept in biosafety level 3 housing and provided with standard laboratory diet and water ad libitum.

### Cytotoxicity assay

Cytotoxic effect of SJS was assessed by 3-(4,5-dimethylthiazol-2-yl)-2,5- diphenyltetrazolium bromide (MTT) assay. MDCK cells (2 × 10^5^) were seeded on a 96-well culture plate in MEM with 10% FBS. After overnight culture, cells were treated with different concentration of SJS in MEM. After 24 h incubation at 37 °C, MTT (5 mg/ml) in phosphate buffered saline (PBS) was freshly prepared, 10 μl of MTT solution was added to each well and the plates were incubated at 37 °C for 4 h. The medium was then removed and formazan crystal was dissolved in dimethyl sulfoxide (DMSO) (100 μl/well). Then the absorbance at 570 nm was read by a CLARIOstar multi-mode microplate reader (BMG Labtech, Germany). The 50% toxic concentration (TC_50_) was calculated as the concentration required to decrease 50% of cell viability.

### Cytopathic effect inhibition (CPE) assay

80% confluent MDCK cells in a 96-well plate were infected with 0.01 MOI of influenza virus for 1 h at 37 °C. Afterwards, the viral inoculum was removed, and cells were washed twice with PBS. 100 μl SJS at different concentration in serum-free MEM with 1 μg/ml TPCK treated-trypsin (TPCK treated-trypsin was absent when MDCK cells were infected by WSN virus) was added to the cells. After incubating at 37 °C for 48 h, 10 μl of 5 mg/ml fresh MTT solution in PBS was added to each well and the plates were incubated at 37 °C for 4 h. The medium was then removed and formazan crystal was dissolved in DMSO (100 μl/well). Absorbance at 570 nm was read by a CLARIOstar multi-mode microplate reader (BMG Labtech, Germany). The concentration that inhibited 50% of virus-induced cytopathic effect was determined as IC_50_.

### Plaque reduction assay

Confluent MDCK cells were seeded in 6-well plates in MEM with 10% FBS. Cells were infected with around 200 pfu per well of different viral strains for 1 h at 37 °C. The inoculum was aspirated to remove unbound viral particles, followed by washing with PBS. The MDCK monolayer was then overlaid with 1% low melting agarose (Cambrex) in MEM which contained different concentration of SJS and 1 μg/ml of TPCK treated-trypsin (TPCK treated-trypsin was absent when MDCK cells were infected by WSN virus). After incubating for 72 h at 37 °C, the agarose was removed and the cell monolayers were stained with staining solution (0.25% coomassie blue, 10% acetic acid, 50% methanol). The number of plaques was counted and the percentage of plaque inhibition relative to the control (no drug treatment) was calculated.

### Multicycle growth assay

80% confluent MDCK cells were seeded in a 24-well plate. After infecting with 0.001 MOI of WSN for 1 h at 37 °C, the inoculum was removed and 500 μl of SJS (500 μg/ml, 125 μg/ml, 60 μg/ml) or oseltamivir (100 μM) in MEM, or 500 μl MEM only were added to the cells and incubated at 37 °C. The supernatants were then collected at 12, 24, 48 and 72 h post infection. The virus titers were determined by plaque assay as described previously [17].

### Hemagglutination inhibition assay

Twofold serial dilution of SJS was prepared in 25 μl of PBS in a 96-well U-bottom plate. WSN in 25 μl of PBS (4 HA units) was added to each dilution and mixed well, and the plate was incubated for 30 min at room temperature. Then, 50 μl of chicken erythrocytes in PBS (0.05% *v*/v) was added to each well and mixed thoroughly. The reaction was observed after incubating the plates at room temperature for another 30 min. Pentagalloyglucose (PGG) was used as a positive control [18] while oseltamivir was a negative control [19].

### Ribonucleoprotein (RNP) reconstitution assay

2 × 10^6^ of 293 T cells were seeded on a 6 cm dish and incubated overnight in DMEM with 10% FBS. Plasmids pcDNA3a-PB1, pcDNA3a-PB2, pcDNA3a-PA, pcDNA3a-NP, pPOL-NS-Luci (kindly provided by Dr. Ervin Fodor, University of Oxford, UK) were transfected to 293 T cells with Lipofectamine 2000 (Invitrogen, CA, USA) to reconstitute the RNP complex. The RNP complex consisted of WSN polymerase proteins PA, PB1 and PB2, NP and a luciferase reporter gene. Plasmid pEGFP was also co-transfected to 293 T cells as an internal control to normalize the transfection efficiency. After 6 h of transfection, transfected cells were trypsinized and aliquoted into a 96-well plate. SJS at different concentration dissolved in DMEM was added into each well. After incubating for 24 h at 37 °C, the cell lysates were harvested and the luciferase activity was assayed with a luciferase reporter assay system kit (Promega, No. E1910). The luminescence was read by a CLARIOstar multi-mode microplate reader (BMG Labtech, Germany).

### Neuraminidase (NA) inhibition assay

A neuraminidase inhibitors screen kit was employed to evaluate the inhibition of SJS on the NA enzymatic activity. The assay followed the instruction manual. 70 μl of reaction buffer, 10 μl of NA and 10 μl of SJS at different concentration were well mixed in a black 96-well microplate. After incubating at 37 °C for 2 min, 10 μl of substrate was added into each well, mixed thoroughly and incubated for 1 h. Fluorescence was measured with a CLARIOstar multi-mode microplate reader (BMG Labtech, Germany) at an excitation wavelength of 322 nm and an emission wavelength of 450 nm. Oseltamivir acid [19] was used as a positive control. The NA activity inhibitory percentage was calculated as follow:

$$ \mathrm{NA}\ \mathrm{inhibition}\ \left(\%\right)=\left({\mathrm{F}}_{\mathrm{control}}\hbox{--} {\mathrm{F}}_{\mathrm{SJS}}\right)/\left({\mathrm{F}}_{\mathrm{control}}\hbox{--} {\mathrm{F}}_{\mathrm{blank}}\right)\times 100\%\left(\mathrm{F}:\mathrm{Fluorescence}\ \mathrm{intensity}\right). $$

### Anti-influenza virus test in mouse model

Mice were randomly divided into vehicle group, SJS group, oseltamivir group and untreated group, with 16 mice in each group. Except the vehicle group, other groups were anaesthetized with ethyl ether and inoculated intranasally with 3 LD_50_ (50% lethal dose) of mouse-adapted PR8 virus in a volume of 50 μl. At 4 h after inoculation, SJS group and oseltamivir group were treated by gavage feeding with SJS solution (dissolved in water at a dose of 1 g/kg/day) or oseltamivir solution (dissolved in water at a dose of 90 mg/kg/day) in a volume of 200 μl, respectively. Then these two groups were administrated orally once daily for seven consecutive days. Untreated group and vehicle group were fed with water. Parameters of mice such as mortality, body weight and general conditions were monitered for consecutive 14 days.

Three mice from each group were randomly selected and sacrificed on the fourth day post-inoculation for lung index calculation, lung viral load titer and lung cytokine expression analysis. Another three mice from each group were also sacrificed on the sixth day post-inoculation for histopathologic observation. The sacrificed mice were euthanized by cervical dislocation after fully anaesthetization by inhaling diethyl ether. The remaining ten mice in each group were monitored continuously for 14 consecutive days to study their mortality and body weight changes.

#### Lung index

Four days after virus infection, mice were weighed and their lung tissues were extracted and washed with PBS, dried by gauze and then weighed. Lung index was calculated as follow:

$$ \mathrm{Lung}\ \mathrm{index}=\mathrm{lung}\ \mathrm{weight}/\mathrm{body}\ \mathrm{weight}\times 100\%. $$

#### Lung viral load titer

After weighing the lung tissues, they were homogenized in MEM by a refiner (Qiagen, TissueRuptor) and centrifuged at 12000 rpm for 5 min at 4 °C. The lung homogenates were aliquoted and stored at − 80 °C. The virus titer of these homogenates were determined by plaque assay [17] on MDCK cells.

#### Lung cytokine expression analysis

Part of the lung homogenates was used to perform lung cytokine expression analysis with mouse cytokines Elisa kits of TNF-α, IFN-α and IL-2 (Invitrogen). The content of TNF-α, IFN-α and IL-2 were assessed according to the manufacturer’s protocol. Absorbance at 450 nm was read by a spectro-photometer (Thermo Scentific).

#### Histopathologic observation

Six days after virus infection, lung tissues were extracted from three randomly sacrificed mice from each group. The lungs were immersed in 10% formaldehyde solution immediately and embedded in paraffin. Then the lung tissue was cut into 4 μm-thick sections. Tissue sections were stained with hematoxylin and eosin for observating the histopathologic changes under microscope.

### Statistics

All statistical analyses were conducted with Graphpad Prism 6.0 (Graphpad, San Diego, CA, USA) and data were presented as mean ± SD. One-way ANOVA was used for multiple-group comparison. Differences were considered statistically significant when *p* < 0.05 (*p < 0.05, **p < 0.01, ***p < 0.001).

## Results

### Anti-influenza activity of SJS against multiple virus strains

**Fig. 1:**
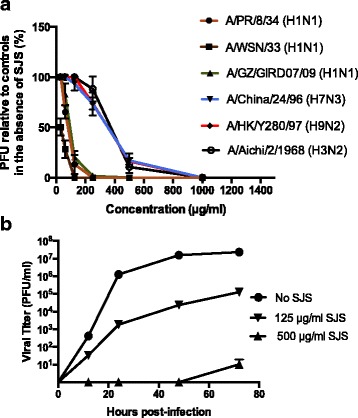
Antiviral activity of SJS in cell culture (**a**) SJS inhibited seven influenza viruses on MDCK cells in plaque reduction assay. Oseltamivir at 100 μM stopped viral growth but data was omitted for clarity. **b** Antiviral effect of SJS in multicycle growth assay. MDCK cells were infected with WSN at MOI = 0.001 in the presence or absence of SJS and the viral progeny at 12, 24, 48 and 72 h were determined by plaque assay. SJS at 1 mg/ml and oseltamivir at 100 μM inhibited viral growth till 72 h. The experiments were carried out in duplicate and repeated three times for confirmation

To determine the inhibitory activity of SJS against the cytopathic effect induced by different viral strains, CPE was performed. Plaque reduction assay was also performed to confirm the antiviral efficacy of SJS on A/WSN/33 (H1N1), A/PR/8/34 (H1N1), A/GZ/GIRD07/09 (H1N1), A/Aichi/2/1968 (H3N2), A/HK/Y280/97 (H9N2) and A/China/24/96 (H7N3). B/Lee/1940 (Flu B) was only tested by CPE assay. The IC_50_ on each strain was calculated based on the results of CPE assay and is shown in Table 1. SJS had a TC_50_ > 2 mg/ml measured by cytotoxicity assay. The selective index (SI) of each strain was also calculated and shown in Table 1. The IC_50_ was ranging from 34.7 to 750.8 μg/ml, and SI was ranging from 2.7 to 57.7. SJS showed the best inhibitory effect on WSN virus (IC_50_ = 34.7 and SI = 57.7). According to the results of plaque reduction assay, SJS inhibited the virus strains in a dose-dependent manner. It inhibited the growth of all the seven viruses (200 pfu per well) to 100% at less than 1 mg/ml (Fig. 1a).

**Table 1:** Antiviral activity of SJS against different influenza virus strain

Viral strains	IC_50_^a^ (μg/ml)	SI^b^ (>)
A/PR/8/34 (H1N1)	198.47	10.08
A/WSN/33 (H1N1)	34.66	57.70
A/GZ/GIRD07/09 (H1N1)	78.56	25.46
A/China/24/96 (H7N3)	371.13	5.39
A/HK/Y280/97 (H9N2)	353.55	5.66
A/Aichi/2/1968 (H3N2)	360.73	5.54
B/Lee/1940 (Flu B)	750.79	2.66

^a^Mean of the results from three independent experiments

^b^The SI (selectivity index) was calculated as the ratio of TC_50_ to IC_50_

To evaluate the influence of SJS on viral progeny, MDCK cells were treated with SJS at 125 μg/ml and 500 μg/ml after infected with WSN virus (MOI = 0.001). The supernatant with replicated virus was collected at 12, 24, 48 and 72 h, and the viral titer at each time point was measured by plaque assay. As shown in Fig. 1b, SJS severely suppressed the WSN viral multicycle growth at 500 μg/ml. On the other hand, oseltamivir as a positive control inhibited viral production at 200 μM at 72 h. This indicated that SJS had a pronounced effect on suppressing the WSN growth.

### SJS did not act on HA and RNP complex

**Fig. 2:**
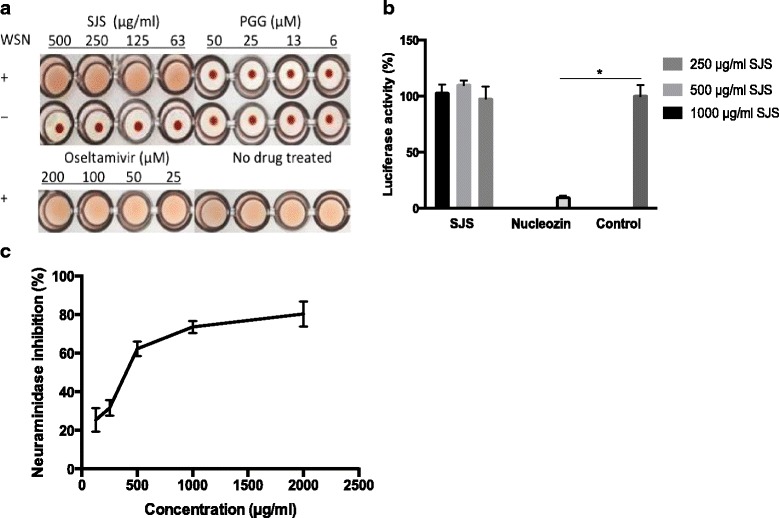
**a** Effect of SJS on hemagglutination with hemagglutinin and chicken erythrocytes. WSN virus (4 HA units) were mixed with 63–500 μg/ml of SJS, and then incubated with 0.05% erythrocytes. Pentagalloyglucose and oseltamivir were used as positive and negative control, respectively. **b** Influence of SJS on viral polymerase complex. 250, 500 and 1000 μg/ml of SJS was added to 293 T cells transfected with WSN minigenomes. Nucleozin at 1 μM was used as a positive control and it inhibited luciferase activity dramatically compared to the control (no drug treated). **c** SJS had an effect on NA activity. Oseltamivir acid was used as a positive control. Under the condition of this kit, oseltamivir acid had its IC_50_at 250 μM. The experiments were carried out in triplicate and repeated three times for confirmation

To determine whether SJS could inhibit hemagglutinin of virus particles for binding to cell surface receptors, hemagglutination inhibition assay was performed. Influenza virus can agglutinate erythrocytes by means of hemagglutination, then erythrocytes become cross-linked and form lattice. In this assay, chicken erythrocytes showed a lattice appearance when treated with SJS at 63–500 μg/ml (in two-fold serial dilution) in the presence of WSN virus (4 HA units) (Fig. 2a). Chicken erythrocytes treated with the positive control PGG at 6–50 μM and WSN virus showed red spot like appearance, indicating the inhibition of hamagglutination. Oseltamivir, on the other hand, had no effect on HA. When WSN was absent, SJS and PGG treatment also showed a red spot appearance, which indicated they had no influence to chicken erythrocytes.

Influenza RNP reconstitution assay was performed to evaluate if SJS inhibited the viral RNP activity. SJS were added to 293 T cells transfected with WSN minigenomes to 250, 500 and 1000 μg/ml. Luciferase activity was measured after 24 h post-transfection. Nucleozin [16], an NP inhibitor, was used as a positive control in the assay. As shown in Fig. 2b, the luciferase activity in the presence of SJS had no obvious differences with the untreated control, while nucleozin inhibited the luciferase activity significantly.

Thus, SJS did not interfere with the absorption of WSN towards the target cells or viral polymerase activity.

### SJS inhibited NA activity

To explore if SJS affected NA enzymatic activity, NA inhibitory assay was conducted using a commercial neuraminidase inhibitors screen kit. Two-fold serial dilutions from 125 μg/ml to 2000 μg/ml of SJS extract were tested. NA activity was measured by fluorescence of 4-methylumbelliferone, which was the product of substrate (4-Methylumbelliferyl)-a-D-N-acetylneuraminic acid sodium salt hydrate catalyzed by the enzymatic activity of NA. Results showed that at 2 mg/ml of SJS, NA activity was inhibited up to 80% (Fig. 2(c)). Oseltamivir acid was used as a positive control and it inhibited 50% NA activity at 250 μM.

### SJS improved survival rate of PR8-infected mice

**Fig. 3:**
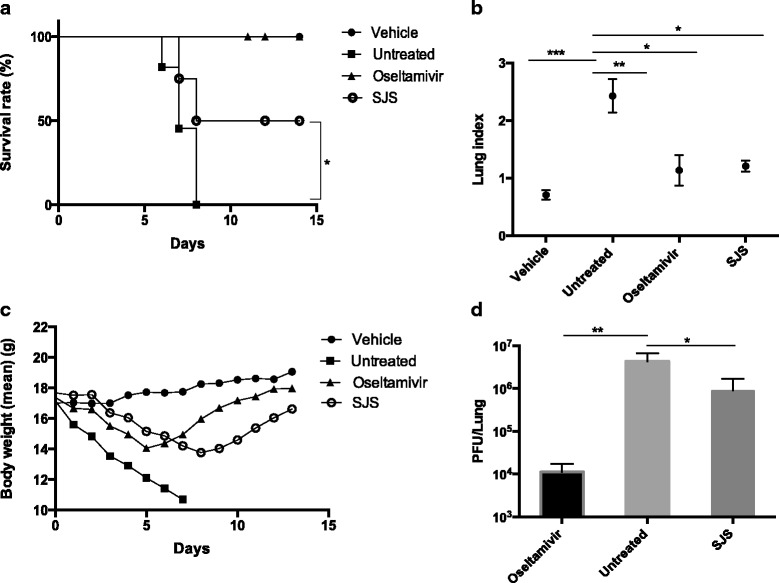
Efficacy of SJS in PR8 infected mouse model. Mice was administrated orally with 200 μl of 1 g/kg/day SJS, 90 mg/kg/d oseltamivir or water for 7 days. Conditions of mice were monitored for consecutive 14 days. **a** SJS protected the mice infected with 3 LD_50_ of PR8 virus. **b** Body weight changes of mice were monitored daily. Oseltamivir and SJS reduced **c** lung index and **d** lung viral load of PR8 infected mice compared with untreated mice. Three mice from each group were randomly selected and sacrificed on the fourth day and their lungs were extracted. The lung viral load was determined by plaque assay

To evaluate the in vivo antiviral efficacy of SJS, groups of mice were inoculated with 3 LD_50_ of mouse lung-adapted viral strain PR8 and orally administrated SJS for seven consecutive days, while vehicle group and untreated group were administrated with water instead. Vehicle control group showed normal appearance and behaviors during the 14-day observation. Mice in the untreated group all died before the 8th day post-inoculation of virus. They also showed inactive, ruffled fur and respiratory distress signs. Mice orally administrated with SJS daily had their life span prolonged. This group showed mortality on day 9 post-inoculation and up to 50% mice survived after 14 days (Fig. 3a). Besides, the average body weight of SJS group rebound on day 9 post-inoculation, similar to the positive control (oseltamivir) group (Fig. 3b). These results suggested that SJS at 1 g/kg/day had significant protective effect on mice infected with PR8 virus.

### SJS reduced lung index, lung viral load and alleviated lung histopathologic changes of PR8 virus infected mice.

Three mice from each group were euthanized on day 4 post-inoculation and their lungs were extracted for lung index measurement and lung viral load titer. Compared with the untreated group, SJS at 1 g/kg/day significantly decreased the mice lung index (Fig. 3c) and inhibited the lung viral load (Fig. 3d).

**Fig. 4:**
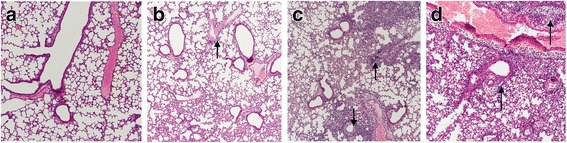
Lung hisotopathologic changes of mice were examined on the sixth day. Representative histologic sections of sacrificed mice from **a** Vehicle group **b** Oseltamivir group **c** SJS group **d** Untreated group were visualized using hematoxylin and eosin staining. The black arrows in B-D highlight the area of inflammatory cell infiltration in submucosal layer of airway epithelium and perivascular region. (Magnification: 100×)

Another three mice were also euthanized on the sixth day post-inoculation for observating the histopathologic changes. As shown in Fig. 4, untreated group showed pronounced lung inflammation, characterized by interstitial expansion, edema and inflammatory cell infiltration around small vessels. Inflammation cells could be observed in alveolar lumen. For SJS group, the histopathology was alleviated and mild lesions were observed. Less inflammatory cells were exuded and infiltrated around vessels and interstitial space. The results of lung index and lung viral load following SJS treatment indicated that SJS treatment alleviated lung pathology and lesion of PR8 infected mice.

### Effect of SJS on lung cytokine expression

**Fig. 5:**
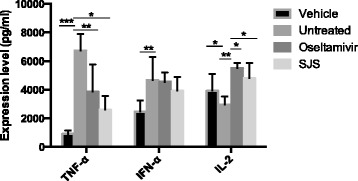
Influence of SJS on cytokine expression in mouse lung infected with PR8 virus. The cytokine expression level of TNF-α, IFN-α and IL-2 was analyzed using Elisa. The experiments were carried out in triplicate and repeated three times for confirmation

To determine the inflammatory markers after SJS treatment, part of the lung homogenates collected on day 4 post-inoculation was used for Elisa assay on the level of cytokine TNF-α, IFN-α and IL-2 (Fig. 5). For the former two markers, their expression in infected group increased significantly compared with the vehicle group. Oseltamivir and SJS treatment both decreased the expression level of these two cytokines after PR8 infection, though IFN-α was only slightly reduced. Cytokine IL-2 in mice lung decreased after PR8 infection, oseltamivir and SJS displayed the same tendency that they increased the level of IL-2. In this Elisa analysis, SJS also showed better regulatory activity on TNF-α and IFN-α than oseltamivir group. These results showed that SJS could reduce the inflammatory responses in mice.

## Discussion

SJS is a classical Chinese formulation for treating influenza virus infection. However, there has been no systematic study to substantiate the anti-influenza efficacy. The mechanism of SJS action also remained unclear.

We first found that SJS could inhibit flu A and B strains on MDCK cell line at less than 750 μg/ml, showing that SJS has a broad-spectrum inhibitory activity to influenza viruses. We also showed that SJS could protect mice from PR8 infection. Compared with the untreated group, SJS could significantly improve the survival rate and prolong the average survival day, also help the body weight of the mice to rebound. SJS treated mice not only had a lower lung viral load but also had reduced lung index and alleviated histopathology, suggesting that SJS might work on the virus directly and SJS could also decrease the lung injury induced by PR8 infection. Compared with oseltamivir, SJS is less effective in protecting PR8 infected mice. However, considering that the formulation containing many phytochemicals, with each in a minute quantity, there is a high chance to have some potent fractions or phytochemicals in the extract.

Previous reports pointed out that over-expression of TNF-α and IFN-α induced by influenza virus is a cause of lung inflammation and can in turn result in tissue injury [20, 21]. In contrast, IL-2 decreases after infected by influenza virus and lower expression of IL-2 causes dysfunction of immune system [11]. In this study, the expression level of three cytokines (TNF-α, IFN-α, IL-2) after PR8 virus infection was examined. Our data showed SJS was beneficial to PR8 infected mice, as SJS could down-regulate TNF-α and up-regulate IL-2. The expression level of IFN-α under treatment of SJS also showed a down-regulatory trend, although there was no statistically significant difference with the untreated group. Meanwhile, compared with the treatment of oseltamivir, SJS showed a stronger regulation on the expression of these cytokines (Fig. 5). Thus, besides inhibiting virus replication directly through targeting on NA, SJS might also protect the mice through regulating the expression of cytokines. We are in the process of carrying out bioassay-guided fractionation to find the pure phytochemicals that inhibit NA and regulate the concerned cytokines.

As influenza infection is a common disease, there are many cold and flu herbal remedies in the market, such as Chinese patent drugs, traditional Chinese prescriptions, or even cooling herbal tea. However, their efficacy in treating influenza is not well investigated. Our research has provided an example on the evidence-based research on an anti-influenza formulation, which involves virus inhibitory studies in cell culture, animal model and mechanism elucidation. The work may be extended to other traditional or folk medicine, which will strengthen the confidence on their clinical uses and for downstream development of these formulations.

## Conclusions

In this study, a Chinese prescription SJS was found to inhibit a number of influenza virus strains and act against influenza virus PR8 in mice. SJS exhibited anti-influenza activity through inhibiting NA activity and regulating cytokine expression. Our work has substantiated that SJS is an effective anti-influenza formulation, which may be further developed by the pharmaceutical industry.

## Additional files

Additional file 1: Method of High-performance liquid chromatography (HPLC) analysis of SJS. HPLC method was used to analyze the chemical profile of SJS. The HPLC condition is described in this additional file and the profile is shown in Additional file 2: Figure S1. By comparing with reference compounds, rhein, chrysophanol, emodin, aloe emodin and curcumin were found. (DOCX 13 kb)

Additional file 2: **Figure S1.** HPLC analysis of SJS (a) HPLC profile of SJS (b) Some constituents were denoted by standard compounds. (PPTX 128 kb)

## Abbreviations

CPE: Cytopathic effect inhibition
DMEM: Dulbecco’s Modified Eagle Medium
DMSO: Dimethyl sulfoxide
Elisa: Enzyme-linked immunosorbent assay
FBS: Fetal bovine serum
HA: Hemagglutinin
IC_50_: 50% inhibitory concentration
IFN: Interferon
IL: Interleukin
LD_50_: 50% lethal dose
MDCK: Madin-Darby canine kidney
MEM: Minimum essential medium
MOI: Multiplicity of infection
MTT: 3-(4,5-dimethylthiazol-2-yl)-2,5- diphenyltetrazolium bromide
NA: Neuraminidase
NP: Nucleoprotein
PA: Polymerase acidic protein
PB1: Polymerase basic protein 1
PB2: Polymerase basic protein 2
PBS: Phosphate buffered saline
Pfu: Plaque forming unit
PGG: Pentagalloyglucose
PR8: A/PR/8/34 (H1N1)
RNP: Ribonucleoprotein
SI: Selective index
SJS: Sheng Jiang San
TC_50_: 50% toxic concentration
TNF: Tumor necrosis factor
TPCK: Tolylsulfonyl phenylalanyl chloromethyl ketone
WSN: A/WSN/33 (H1N1)
