# Supplementary material for: Sheng Jiang San, a traditional multi-herb formulation, exerts anti-influenza effects in vitro and in vivo via neuraminidase inhibition and immune regulation
Source: BMC Complement Altern Med. 2018 May 8;18:150. doi: 10.1186/s12906-018-2216-7 (PMC5941478; doi:10.1186/s12906-018-2216-7)
Supplement: Supplementary file 1 — Method of High-performance liquid chromatography (HPLC) analysis of SJS. HPLC method was used to analyze the chemical profile of SJS. The HPLC condition is described in this additional file and the profile is shown in Additional file 2: Figure S1. By comparing with reference compounds, rhein, chrysophanol, emodin, aloe emodin and curcumin were found. (DOCX 13 kb) [file 12906_2018_2216_MOESM1_ESM.docx]

**Additional file 1**

***Method of High-performance liquid chromatography (HPLC) analysis of SJS***

HPLC analysis was performed by Agilent 1260 HPLC system (Agilent Technologies), which equipped with a degasser, quaternary pump, thermostatted column oven, autosampler and photodiode array detector. The SJS sample was separated on Agilent Zorbax 300SB-C18 column (4.6×250 mm, 5 μm) at 40 °C. The mobile phase system was composed of (A) water with 0.2% acetic acid and (B) methanol. The HPLC gradient elution profile was: 0-2 min, 10%B; 2-4 min, 45%B; 4-20 min, 50%B; 20-30min, 55%B; 30-40min, 70%B; 40-44min, 70%B; 44-47min, 10%B; 47-50 min, 10%B. The mobile phase flow rate was 1.0 ml/ml and the UV spectrum was 430 nm. The injection volume was 10 μl. Standard compounds were also analyzed by the same HPLC analysis method. Data acquisition was performed by Agilent ChemStation software (Agilent Technologies).
