# Supplementary figures and images for: Sheng Jiang San, a traditional multi-herb formulation, exerts anti-influenza effects in vitro and in vivo via neuraminidase inhibition and immune regulation
Source: BMC Complement Altern Med. 2018 May 8;18:150. doi: 10.1186/s12906-018-2216-7 (PMC5941478; doi:10.1186/s12906-018-2216-7)

## Slide 1
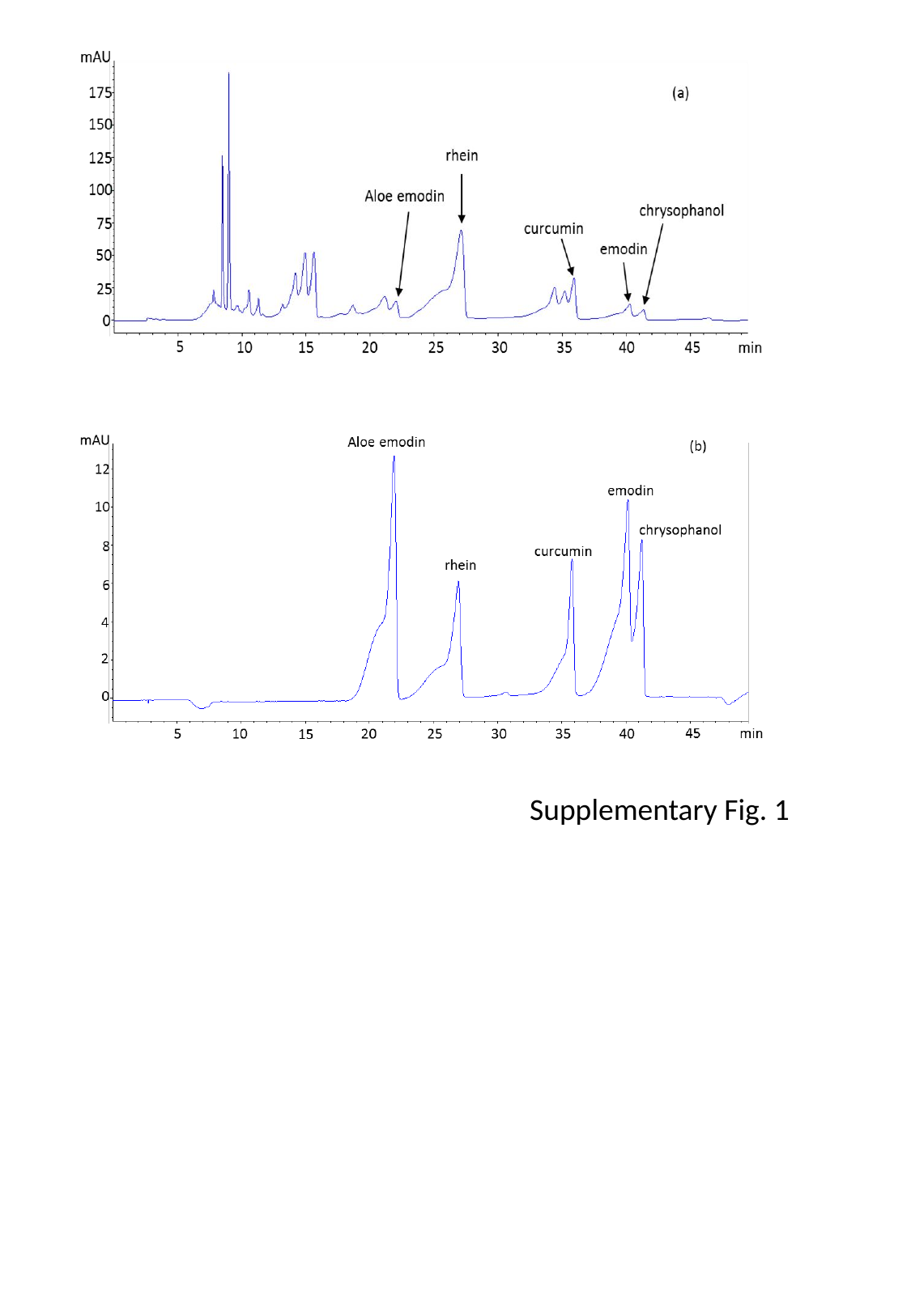

Supplementary Fig. 1

Supplement: Supplementary file 2 — Figure S1. HPLC analysis of SJS (a) HPLC profile of SJS (b) Some constituents were denoted by standard compounds. (PPTX 128 kb) [file 12906_2018_2216_MOESM2_ESM.pptx]
